# Polydopamine–polyethylene glycol–liproxstatin-1 nanoparticles inhibit ferroptosis for enhanced treatment of neutrophilic asthma

**DOI:** 10.3389/fphar.2026.1809896

**Published:** 2026-06-11

**Authors:** Chen Bao, Decai Wang, Chao Liu, Meizhou Zhang, Qian Liu, Jiannan Hu, Yuzhou Wu, Shuyun Xu

**Affiliations:** 1 Department of Pulmonary and Critical Care Medicine, Shanghai Geriatric Medical Center, Shanghai, China; 2 Department of Pulmonary and Critical Care Medicine, Zhongshan Hospital, Fudan University, Shanghai, China; 3 Shanghai Respiratory Research Institute, Shanghai, China; 4 Department of Respiratory and Critical Care Medicine, Tongji Hospital, Tongji Medical College, Huazhong University of Science and Technology, Wuhan, Hubei, China; 5 Key Laboratory of Respiratory Diseases of National Health Commission, Tongji Hospital, Tongji Medical College, Huazhong University of Science and Technology, Wuhan, Hubei, China; 6 School of Life Science and Technology, Pilot Base of Food Microbial Resources Utilization of Hubei Province, Wuhan Polytechnic University, Wuhan, Hubei, China; 7 Hubei Key Laboratory of Bioinorganic Chemistry and Materia Medica, Key Laboratory of Material Chemistry for Energy Conversion and Storage, Ministry of Education, School of Chemistry and Chemical Engineering, Hubei Engineering Research Center for Biomaterials and Medical Protective Materials, Huazhong University of Science and Technology, Wuhan, Hubei, China

**Keywords:** asthma, ferroptosis, lipid peroxidation, liproxstatin-1 (LIP-1), nanoparticles

## Abstract

Ferroptosis is an iron-dependent programmed cell death mechanism triggered by the accumulation of lipid-based reactive oxygen species (ROS). It is closely implicated in the pathogenesis of asthma. Liproxstatin-1 (LIP-1) is a ferroptosis inhibitor that is beneficial for treating neutrophilic asthma. However, low water solubility, limited blood concentration, and poor mucus permeability and biocompatibility limit the therapeutic efficacy of LIP-1. In this study, we successfully constructed polydopamine–polyethylene glycol–LIP-1 nanoparticles (PDA-PEG-LIP-1 NPs) for the treatment of neutrophilic asthma. Inhalation of PDA-PEG-LIP-1 NPs effectively inhibited lipopolysaccharide (LPS)- and interleukin (IL)-13-induced ferroptosis by alleviating lipid peroxidation and ROS production and chelating free ferrous ions (Fe^2+^). In addition, results from asthma mouse models demonstrated that inhalation of PDA-PEG-LIP-1 NPs could overcome the limitations of LIP-1 and effectively inhibit ferroptosis. This research suggests that PDA-PEG-LIP-1 NPs are an effective, practical, and safe option for treating neutrophilic asthma.

## 1 Introduction

Neutrophilic asthma is characterized by airway neutrophilic inflammation and is closely associated with severe asthma. It is insensitive to conventional corticosteroid therapy and therefore poses a significant threat to human health. However, the specific pathogenesis of neutrophilic asthma remains poorly elucidated, and effective therapeutic strategies are still lacking. Consequently, there is a pressing need to identify novel agents that target the symptoms and phenotypes of neutrophilic asthma (Hammad et al., 2025).

A distinct form of cell death known as ferroptosis is characterized by the accumulation of iron-dependent lipid reactive oxygen species (ROS). According to recent research, ferroptosis plays a crucial role in the pathogenesis of asthma (Wientjens et al., 2025; Chen et al., 2025). As a small-molecule lipophilic antioxidant, liproxstatin-1 (LIP-1) exerts anti-ferroptotic effects by suppressing the accumulation of lipid hydroperoxides (Zilka et al., 2017). We have recently demonstrated that LIP-1 can inhibit ferroptosis in airway epithelial cells and may serve as an effective therapeutic agent for neutrophilic asthma (Bao et al., 2022). Despite its therapeutic potential, LIP-1 has limited efficacy in treating neutrophilic asthma due to its poor water solubility, low blood concentration, weak mucus penetration, and unsatisfactory biocompatibility (Zilka et al., 2017). At present, it remains unclear whether these defects of LIP-1 can be ameliorated to develop more efficient drugs for asthma treatment.

Polymer-based nanoparticles (NPs) are widely used to develop drug delivery systems due to their ideal biocompatibility and ability to optimize the pharmacokinetic properties of specific drugs (Beach et al., 2024). Polydopamine (PDA), formed by the self-polymerization of dopamine, exhibits unique chemical properties and has attracted substantial attention in nanomedicine. Because PDA NPs can adsorb diverse molecules, they are widely used for drug delivery, molecular imaging, and cancer therapy (Acter et al., 2023; Bedhiafi et al., 2023; Jin et al., 2020; Li et al., 2021). A recent study reported that PDA inhibited ischemia/reperfusion (I/R) injury-induced ferroptosis in cardiomyocytes (Zhang et al., 2021). The abundant phenolic hydroxyl groups on PDA enable efficient chelation of metal ions including iron, copper and magnesium, which is crucial for inhibiting ferroptosis (Zhang et al., 2021; Jiang et al., 2025). Moreover, due to the presence of numerous phenolic groups, PDA can scavenge ROS and has been used to alleviate ROS-mediated injury and inflammation (Yang et al., 2023; Bao et al., 2023).

Goblet cells in the airway secrete large amounts of mucus. Traditional inhaled drugs fail to efficiently penetrate the mucus barrier to reach epithelial cells and are prone to phagocytosis by macrophages. Polyethylene glycol (PEG) modification can endow NPs with electrically neutral, hydrophilic surfaces, which weakens electrostatic and hydrophobic interactions between NPs and mucus components. This property facilitates nanoparticles penetration across the airway mucus barrier and prolongs the *in vivo* circulation half-life of drugs (Poinard et al., 2019; D'Souza A and Shegokar, 2016; He et al., 2024).

In this study, we developed LIP-1-loaded PEG-modified PDA (PDA-PEG-LIP-1) NPs as a preventive strategy to alleviate OVA- and LPS-induced neutrophilic asthma *in vivo*. We systematically evaluated the therapeutic potential and underlying molecular mechanisms of PDA-PEG-LIP-1 NPs, with particular focus on its anti-inflammatory and anti-ferroptotic activities in cellular and murine models. It is anticipated that these findings will provide an experimental basis and theoretical support for the development of novel targeted therapeutic strategies targeting the airway epithelial cells of neutrophilic asthma.

## 2 Materials and methods

### 2.1 Materials

Dopamine hydrochloride (DA; Cat #: E080933) was obtained from Energy Chemical Co., Ltd. (Shanghai, China). Aqueous ammonia (NH_3_·H_2_O, 28%; Cat #: 10002118) and absolute ethyl alcohol (Cat #: 10009218) were purchased from Hushi Reagent Co., Ltd. (Shanghai, China). Methoxyethylene glycol amine (mPEG-NH2; MW = 2000Da; Cat #: A163261) and iron (II) sulfate hydrate (FeSO_4_·xH_2_O; Cat #: F302448) were obtained from Aladdin Reagent Co., Ltd. (Shanghai, China). Liproxstatin-1 (LIP-1; Cat #: HY-12726), fluorescein 5-isothiocyanate (FITC; Cat #: HY-66019), and 2′,7′-dichlorodihydrofluorescein diacetate (DCFH-DA; Cat #: HY-D0940) were purchased from Med Chem Express Co., Ltd. (Shanghai, China). Simulated lung fluid (Cat #: Chemazone469) was purchased from Chemazone Inc. C11-BODIPY (Cat #: RM02821) was purchased from Abclonal Co., Ltd. (Wuhan, China). FerroOrange (Cat #: F374) was purchased from Dojindo Co., Ltd. (Japan). Cell Counting Kit-8 The Total Antioxidant Capacity (T-AOC) Assay Kit (DPPH; Cat #: D799296) was purchased from Sangon Co., Ltd. (Shanghai, China). The Total Superoxide Dismutase (SOD) Assay Kit (Cat #: S0109) and Lipid Peroxidation MDA Assay Kit (Cat #: S0131S) were purchased from Beyotime Institute of Biotechnology Co., Ltd. (Shanghai, China). The Iron Assay Kit (Cat #: TC1015) was purchased from Leagene Biotechnology Co., Ltd. (Beijing, China). The Dihydroethidium (DHE) Assay Kit (Cat #:ab236206) was purchased from Abcam Co., Ltd. (UK). Multisciences Co., Ltd. (Hangzhou, China) provided ELISA kits. LPS (Cat #: L2880) and OVA (grade V, Cat #: A5503; grade II, Cat #: A5253) were purchased from Sigma-Aldrich (USA). IL-13 (Cat #: 200-13) was purchased from PeproTech (USA). Aluminum hydroxide (Cat #: 77161), Dulbecco’s modified Eagle medium (DMEM; Cat #: 11995040), and fetal bovine serum (FBS; Cat #: 10099141) were acquired from Thermo Fisher Scientific Co., Ltd. (USA). ATCC (Manassas, VA, USA) supplied the human bronchial epithelial cells (16HBE). Female C57BL/6J mice were obtained from Charles River Co. Ltd. (Beijing, China).

### 2.2 Synthesis of PDA-PEG-LIP-1 NPs

First, PDA NPs were synthesized using previously reported methods with minor modifications (Lu et al., 2019; Liu et al., 2013). In brief, deionized (DI) water (9 mL) and ethanol (4 mL) were mixed with aqueous ammonia (0.33 mL) and blended for 30 min. The mixture was then added to DA (1 mL, 50 mg/mL) and stirred for 24 h at 30 °C. The resulting solution was washed three times with deionized water. The amount of PDA NPs was determined by weight lyophilization.

Next, PEGylated PDA NPs were synthesized. The pH of the PDA NPs (2 mg/mL) aqueous solution was adjusted to 9.0 using aqueous ammonia. PDA NPs (5 mL) were mixed with mPEG_2k_-NH_2_ (2 mg/mL) and reacted overnight. The mixture was filtered three times with deionized water the following day and centrifuged at 12,000 rpm to obtain the crude product(Zhang et al., 2021).

LIP-1 was then loaded onto the surface of PEGylated PDA NPs. LIP-1 (30 μg) was dissolved in 15 mL of H_2_O-DMSO (14:1). This solution (2 g/mL, 15 mL) was added to the aqueous solution of PEGylated PDA NPs (1 mg/mL, 15 mL) obtained in the previous step. After magnetic stirring overnight, the mixture was centrifuged to remove free LIP-1, and the precipitated NPs were resuspended in water. Then, PDA-PEG-LIP-1 NPs were finally obtained via freeze-drying.

### 2.3 Characterization of PDA-PEG-LIP-1 NPs

All synthesized NPs were characterized using the Hitachi 7700 (Japan) transmission electron microscope operating at 100 KV and the Hitachi SU8010 (Japan) scanning electron microscope operating at 3 KV. Dynamic light scattering (DLS; Malvern Mastersizer 2000, UK) was used to measure the zeta potential, particle size and polydispersity index (PDI) of the NPs. Fourier-transform infrared (FTIR) spectroscopy (Bruker Vertex 70, Germany) was used to evaluate NP transmittance.

### 2.4 LIP-1 entrapment efficiency of PDA-PEG-LIP-1 NPs

Thermo Fisher Scientific’s Varioskan LUX automatic microplate reader was utilized to identify the distinctive UV absorption peak of LIP-1. The LIP-1 concentration in PEGylated PDA NPs was assessed using the obtained UV spectra. A standard calibration curve was plotted to quantify the concentration of loaded LIP-1. PEGylated PDA NPs (1 mg/mL) were mixed with different concentrations of LIP-1 (0.5 μg/mL, 1 µg/mL, 2 μg/mL, 5 μg/mL, and 10 μg/mL) at equal volumes, and the mixture was stirred in the dark for 24 h. Centrifugation was used to separate the free LIP-1, and the entrapment efficiency (EE) was determined through the following formula:

EE=mass of loaded drugs/total mass of fed drugs×100%

### 2.5 Determination of the drug release capacity of PDA-PEG-LIP-1 NPs

LIP-1 *in vitro* release was measured by dynamic dialysis at 37 °C in phosphate buffer (pH = 7.3). The amount of LIP-1 released at different time intervals was recorded by UV spectroscopy.

### 2.6 Evaluation of PDA-PEG-LIP-1 NPs’ iron-chelating capacity

PDA-PEG-LIP-1 NPs were incubated with 0-, 10-, 100-, 200-, and 500 μM Fe^2+^ at room temperature for 4 h to test their iron-chelating capacity. The NPs were recovered by centrifugation at 12,000 rpm for 20 min and filtered three times with deionized water to remove unchelated Fe^2+^. The zeta potential of NPs dispersed in deionized water was measured by DLS.

### 2.7 Analysis of the PDA-PEG-LIP-1 NPs’ capacity to scavenge ROS

The DPPH radical-scavenging ability of PDA-PEG-LIP-1 NPs was examined according to a previously reported method, with appropriate modifications (Tian et al., 2022). Briefly, 20 μL of PDA-PEG-LIP-1 NP sample (double distilled water) at different concentrations (0, 12.5, 25, 50, 100, and 200 μg/mL) was added to 380 μL of the DPPH reagent and vortexed in the dark for 20 min at room temperature. Absorbance was measured at 515 nm, and the inhibition rate was calculated using the following equation:

$$\%\text{DPPH}\cdot \text{ scavenged}=\left[\frac{\left(A_{0}-A_{1}\right)}{A_{0}}\right]\times 100\%$$

A_1_ represents the absorbance of the sample, A_0_ represents the absorbance of the control solvent, and A_2_ represents the absorbance of the blank reagent without sodium salicylate in the above equation.

Using the Nitro Blue Tetrazolium (NBT) and total Superoxide Dismutase (SOD) Assay Kit technique, the superoxide anion (O_2_^·-^)-scavenging capacity of PDA-PEG-LIP-1 NPs was evaluated (Bao et al., 2018). Briefly, the NBT/enzyme working solution, reaction-starting solution, and NP samples at different concentrations (0, 12.5, 25, 50, 100, and 200 μg/mL) were mixed. On a microplate reader, absorbance at 560 nm was determined after 30 min of incubation at 37 °C in the dark. The inhibition rate was calculated according to the operation instructuons.

A recent study investigated the hydroxyl radical (•OH)-scavenging capacity of PDA-PEG-LIP-1 NPs(Tian et al., 2022). In summary, 100 μL each of sodium salicylate (9 mM), FeSO_4_ (9 mM), and H_2_O_2_ were mixed with 150 μL of PDA-PEG-LIP-1 NPs at different concentrations (0, 12.5, 25, 50, 100, and 200 μg/mL). After incubation at 37 °C for 30 min, absorbance was measured at 510 nm, and the inhibition rate was calculated using the following equation:

$$\%\cdot \text{OH scavenged}=\left[1-\frac{\left(A_{1}-A_{2}\right)}{A_{0}}\right]\times 100\%$$

A_1_ represents the absorbance of the sample, A_0_ represents the absorbance of the control solvent, and A_2_ represents the absorbance of the blank reagent without sodium salicylate in the above equation.

### 2.8 *In vitro* cytotoxicity and cell uptake of PDA-PEG-LIP-1 NPs

HBE cells were seeded in 96-well plates and cultured in DMEM supplemented with 10% FBS at 37 °C and 5% CO_2_ for 12 h to evaluate the cytotoxicity of PDA-PEG-LIP-1 NPs. The cells were treated with PDA-PEG-LIP-1 NPs at concentrations of 0, 1, 5, 10, 20, 50, 100, 200, and 500 μg/mL for 24 h, or 100 μg/mL for 0, 2, 4, 6, 12, 24, 36, 48, and 72 h. Cell viability was then measured using the CCK-8 kit assay.

To evaluate cellular uptake, PDA-PEG-LIP-1 NPs (1 mg/mL) were incubated with FITC (1 mg/mL) for 12 h and then rinsed repeatedly with PBS. HBE cells were seeded in 6-well plates and incubated with PDA-PEG-LIP-1-FITC NPs at concentrations of 0, 1, 5, 10, 20, 50, and 100 μg/mL for 4 h to observe the cell uptake of NPs at different concentrations. For time-dependent uptake analysis, the cells were treated with 100 μg/mL PDA-PEG-LIP-1-FITC NPs for 0, 1, 2, 4, 6, 8, 12, and 24 h. The cells were washed three times with PBS and stained with DAPI to label nuclei. After cleaning with PBS, the cells were photographed using an Olympus FV3000 confocal laser scanning microscope (Japan). In addition, a flow cytometer (CytoFLEX S, Beckman Coulter, USA) was used to quantify FITC-positive HBE cells after treatment with PDA-PEG-LIP-1-FITC NPs.

### 2.9 Assessment of intracellular ROS, lipid peroxide, and Fe^2+^ levels

HBE cells were seeded in 6-well plates at a density of 1 × 10^5^ cells/well and cultured for 12 h to evaluate intracellular ROS levels. The cells were respectively pre-treated with PBS, LIP-1 (1 μM), PDA-PEG NPs (100 μg/mL), or PDA-PEG-LIP-1 NPs (100 μg/mL) for 1 h. Subsequently, the cells were incubated with or without LPS + IL-13 for 24 h to induce oxidative stress. The cells were then cultured in DCFH-DA solution away from light for 30 min during the next day. Finally, fluorescence intensity was measured by a flow cytometer to assess the intracellular ROS levels in each group. To determine intracellular lipid peroxide levels, HBE cells were treated as described above. After 24 h of treatment, the cells were incubated with 2.5 μM C11 BODIPY away from light for 30 min, and fluorescence intensity of each group was measured using a flow cytometer.

The abovementioned treatment was also applied to HBE cells to measure intracellular Fe^2+^ levels. The cells underwent a 24 h treatment period, followed by a 30 min incubation in DMEM with 1 µM FerroOrange. Images were taken using an Olympus IX53 fluorescent microscope (Japan).

HBE cells were seeded in 96-well plates and cultured in DMEM containing 10% FBS for 12 h at 37 °C and 5% CO_2_ to determine the vitality of the cells. The cells were divided into eight groups: control (CON), LIP-1, PDA-PEG, PDA-PEG-LIP-1, LPS + IL-13, LPS + IL-13 + LIP-1, LPS + IL-13 + PDA-PEG and LPS + IL-13 + PDA-PEG-LIP-1 groups. Cells in these groups were treated with PBS, LIP-1 (1 μM), PDA-PEG NPs (100 μg/mL), PDA-PEG-LIP-1 NPs (100 μg/mL), LPS + IL-13 (10 μg/mL and 10 ng/mL, respectively), LPS + IL-13 + LIP-1 (10 μg/mL, 10 ng/mL, and 1 μM, respectively), LPS + IL-13 + PDA-PEG NPs (10 μg/mL, 10 ng/mL and 100 μg/mL, respectively) and LPS + IL-13 + PDA-PEG-LIP-1 NPs (10 μg/mL, 10 ng/mL and 100 μg/mL, respectively) for 24 h, respectively. Cell viability was then evaluated using the CCK-8 kit.

### 2.10 Assessment of the efficacy of PDA-PEG-LIP-1 NPs in treating neutrophilic asthma in mice

The C57BL/6J female mice were housed in an SPF. They were 8 weeks old and weighed approximately 20 g. They were maintained in a controlled environment (22 °C 2 °C, 55% 5% humidity) under a 12 h light/dark cycle and had free access to standard feed and water. Before experimentation, all mice underwent a 1-week quarantine and acclimatization period.

To determine the localization of PDA-PEG-LIP-1 NPs in mouse airway epithelial cells, PDA-PEG-LIP-1 NPs (1 mg/mL) were incubated with FITC (1 mg/mL) for 12 h and then rinsed repeatedly with PBS. The FITC-labelled NPs were delivered directly to the lung via intranasal administration. Mice were killed after 24 h, and their lung tissues were harvested, embedded in OCT, and cryo-sectioned after snap frozen at −80 °C. Airway epithelial cells were labeled with CY3-EpCAM, and nuclei were stained with DAPI. The cells underwent imaging via laser confocal microscopy.

A total of 80 mice were divided into eight groups, each with 10 mice: control (CON), PDA-PEG, PDA-PEG-LIP-1, OVA + LPS, OVA + LPS + LIP-1 intraperitoneal (i.p.) injection, OVA + LPS + LIP-1 intranasal (i.n.) instillation, OVA + LPS + PDA-PEG, and OVA + LPS + PDA-PEG-LIP-1 groups. With some adjustments, neutrophilic asthma was generated as previously described (Lu et al., 2016). On days 0, 7, and 14 of treatment, mice in the OVA + LPS, OVA + LPS + LIP-1 (i.p.), OVA + LPS + LIP-1 (i.n.), OVA + LPS + PDA-PEG and OVA + LPS + PDA-PEG-LIP-1 groups were intraperitoneally injected with 100-μg sensitized OVA (grade V) and 1-mg aluminum hydroxide. In contrast, those in the CON,PDA-PEG and PDA-PEG-LIP-1 groups were injected with an equal volume of saline. On days 21, 23, 25, and 27, mice in the CON and OVA + LPS groups received saline intranasally. Mice in the OVA + LPS + LIP-1 (i.p.) group were intraperitoneally injected with LIP-1 (10 mg/kg), whereas mice in the OVA + LPS + LIP-1 (i.n.) group received LIP-1 (1 mg/kg) intranasally, mice in the OVA + LPS + PDA-PEG group were administered PDA-PEG (1 mg/kg) intranasally and mice in the OVA + LPS + PDA-PEG-LIP-1 and PDA-PEG-LIP-1 groups were administered PDA-PEG-LIP-1 (1 mg/kg) intranasally. Mice in the OVA + LPS, OVA + LPS + LIP-1 (i.p.), OVA + LPS + LIP-1 (i.n.), OVA + LPS + PDA-PEG, and OVA + LPS + PDA-PEG-LIP-1 groups received saline after 1 h of treatment. In contrast, those in the CON, PDA-PEG and PDA-PEG-LIP-1 groups received an aerosol formulation of OVA (3% grade II) as an alternative. All mice were sacrificed on day 28. The procedures for sensitization, treatment, and challenge are illustrated in Figure 7A. Every test was conducted according to established protocols and were approved by the Ethics Committee of Tongji Hospital, Huazhong University of Science and Technology.

For bronchoalveolar lavage fluid (BALF) collection and cell count, the lungs were cleaned three times with 0.7 mL of PBS using a 1 mL syringe with an integrated cannula. BALF was collected and centrifuged, and the supernatant was rapidly frozen to analyze cytokine levels. After resuspension, the cell density was adjusted to 20,000/80uL DMEM. Liu’s solution was created and stained cell slide smears under the manufacturer’s (Promoter Biotech, China) instructions. Differential cell counts were performed blindly to determine the numbers of macrophages, eosinophils, lymphocytes, and neutrophils. A total of 200 cells per slide were counted under a magnification of 400x.

Mouse lung, heart, liver, kidney, and spleen tissues were sectioned at a thickness of 4 μm and stained with hematoxylin and eosin (H&E). H&E-stained sections were evaluated and scored according to a previously described method (Xie et al., 2021). Semiquantitative assessment was performed based on four parameters: perivascular edema (P1), perivascular immune cell recruitment (P2), goblet cells in bronchioles (P3), and macrophages in alveolar spaces (P4). Scoring criteria were as follows: P1 was graded based on the proportion of dilated perivascular spaces surrounding veins: 0, no change; 1, 0%–25%; 2, 25%–75%; 3, >75%. P2 was scored according to the thickness of immune cell infiltration around veins: 0, absent; 1, <3 cell layers; 2, 3–5 cell layers; 3, >5 cell layers. P3 was assessed by counting goblet cells in two bronchiolar profiles: 0, absent; 1, <10 cells; 2, >10 cells. P4 was determined by enumerating eosinophilic macrophages in two alveolar spaces: 0, absent; 1, <10 cells; 2, >10 cells. The total histological score (H-score) was calculated as the sum of P1, P2, P3, and P4. Periodic acid Schiff (PAS) was used to stain lung tissue sections to identify cells that secrete mucus. The abundance of PAS-positive cells in the airway was graded by the following: 0, <5%; 1, 5%–25%; 2, 25%–50%; 3, 50%–75%; 4, >75%. Immunohistochemical staining was performed as described previously (Zhan et al., 2022). Anti-GPX4 antibody (1:1000) was used to analyze lung tissue sections. An Olympus microscope was used for acquiring images. Two independent examiners blinded to the experimental conditions and techniques gave the above grades.

The effects of PDA-PEG-LIP-1 NPs on ROS release in mouse lung tissues were investigated using DHE staining. The detailed experimental protocol was similar to that reported previously (Zheng et al., 2022). Frozen lung sections were restored to room temperature and stained with DHE following the instructions. Sections underwent a 30 min DHE incubation at 37 °C followed by four washes with PBS. The slides were cleaned and dried after the DAPI staining of the cell nuclei. The last step included utilizing an anti-fluorescence quencher and a fluorescence microscope to capture photos. Using ImageJ, the formation of ROS in lung tissues was examined.

To measure the levels of MDA and Fe^2+^, mouse lung tissues were homogenized in an ice bath and centrifuged at 10,000 g/min for 10 min. Proteins in supernatants were measured using a bicinchoninic acid (BCA) assay. Subsequently, the supernatants were used for evaluating MDA and Fe^2+^ levels following the instructions, with absorbance measured at 532 nm and 562 nm, respectively.

Blood samples were collected and centrifuged at 3000 rpm for 15 min to access liver and kidney function. Serum levels of ALT, AST, BUN, and CRE were measured using a biochemical analyzer (Biobase BK280, China).

### 2.11 Assessment of mitochondrial morphology via TEM

To observe mitochondrial morphology, HBE cells or mouse lung tissues were fixed in EM fixative solution at room temperature for 1 h and stored at 4 °C until further processing. Dehydrated, epoxy-embedded fixed cells or tissues were treated at 60 °C for 48 h. The implanted samples were then sectioned into ultrathin slices and stained with uranyl acetate and lead citrate. Images were acquired using a transmission electron microscope.

### 2.12 Western blotting

On 12% sodium dodecyl sulfate–polyacrylamide gels (Sangon Biotech, Shanghai, China), extracted proteins were separated and electroblotted on a PVDF membrane (Roche, UK). Membranes were blocked and incubated overnight at 4 °C with primary antibodies labeled against GAPDH (1:5000; Proteintech, Wuhan, China; Cat #: 10494–1-AP), GPX4 (1:2000; Proteintech, Wuhan, China; 67763–1-Ig), or SLC7A11 (1:1000; Proteintech, Wuhan, China; 26864–1-AP). The following day, membranes were washed with TBST and incubated with horseradish peroxidase-conjugated goat anti-rabbit IgG (1:4000; Proteintech, Wuhan, China; Cat #: SA00001-2) or horseradish peroxidase-conjugated goat anti-mouse IgG (1:4000; Proteintech, Wuhan, China; Cat #: SA00001-1) for 1 h at room temperature.

Protein bands were visualized using a chemiluminescence reagent (Cat #: 1705061; Bio-Rad, USA), and band intensities were quantified using Image-Pro Plus (version 6.0) software. GAPDH was applied as a standard.

### 2.13 ELISA (enzyme-linked immunosorbent assay)

According to the manufacturer’s instructions, ELISA kits were used to measure IL-33, thymic stromal lymphopoietin (TSLP), and CXCL1 levels in BALF. The detection limits for CXCL1, TSLP, and IL-33 were 0.47, 0.45, and 0.26 pg/m, respectively.

### 2.14 Statistical analysis

GraphPad Prism (version 8.0) or SPSS Statistics (version 26.0) (IBM, Armonk, NY, USA) software was utilized for all statistical analyses. Differences between groups were analyzed using one-way ANOVA or Student’s t-test followed by Tukey *post hoc* test (p < 0.05). Data are presented as mean ± standard deviation (±SD) for the control and experimental samples.

## 3 Results

### 3.1 Characterization and synthesis of PDA-PEG-LIP-1 NPs

**FIGURE 1:**
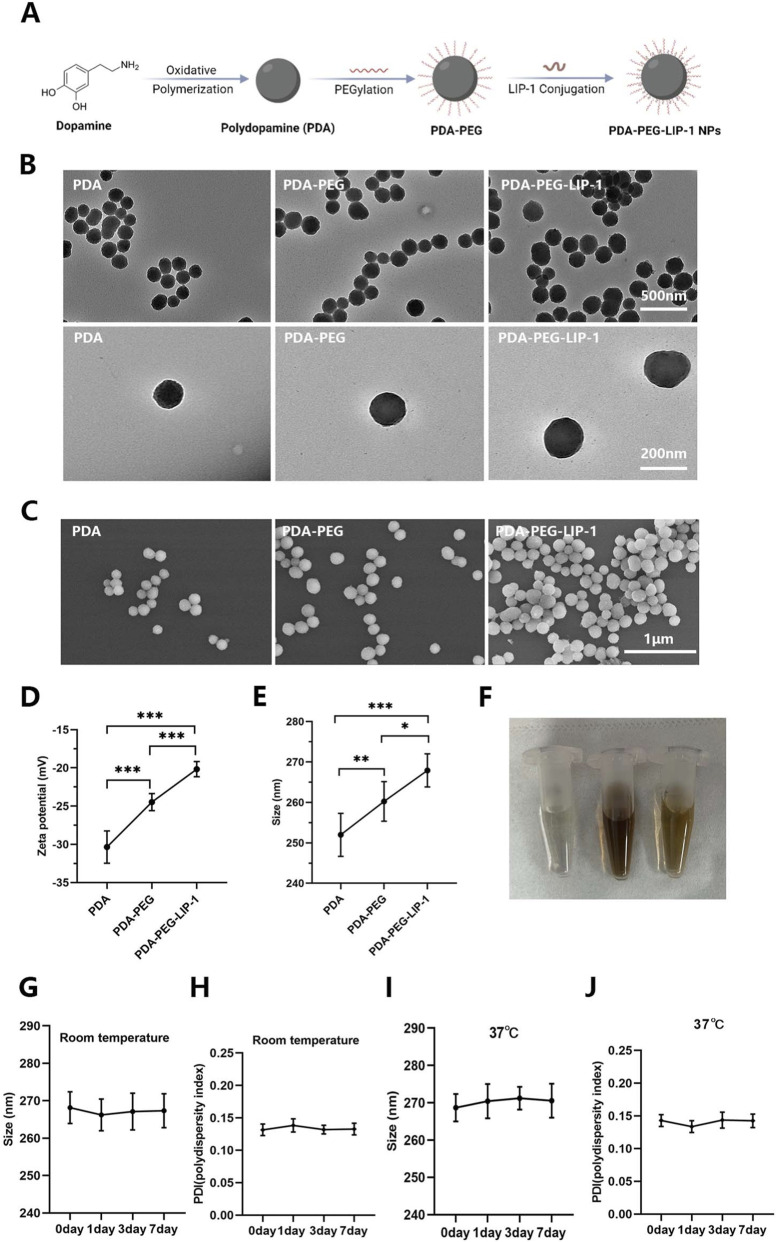
**(A)** Synthesis of PDA-PEG-LIP-1 NPs. **(B)** Transmission Electron Microscopy images of PDA-PEG-LIP-1 NPs, PDA-PEG, and PDA (scale bar = 200 nm and 500 nm). **(C)** SEM images of PDA-PEG-LIP1 NPs, PDA-PEG, and PDA (scale bar = 1 μm). Zeta potential **(D)** and size distribution **(E)** of PDA-PEG-LIP1 NPs, PDA-PEG, and PDA in ultrapure water (n = 6). **(F)** Photograph of the stability of LIP-1 (left), PDA-PEG NPs (middle), and PDA-PEG-LIP-1 NPs (right) after standing for 24 h. Particle size distribution **(G)** and polydispersity index **(H)** of PDA-PEG-LIP-1 NPs in simulated lung fluid over time at room temperature (n = 6). Particle size distribution **(I)** and polydispersity index **(J)** of PDA-PEG-LIP-1 NPs in simulated lung fluid over time at 37 °C(n = 6). Data are expressed as mean ± SD (*, P < 0.05; **, P < 0.01; ***, P < 0.0001).

The method used for synthesizing PDA-PEG-LIP-1 NPs is shown in Figure 1A. To summarize, dopamine was oxidized and auto-polymerized in water, ethanol, and ammonia for 24 h at 30 °C to produce PDA NPs (Lu et al., 2019). Methoxy polyethylene glycol amine (mPEG_2k_-NH_2_) was used to modify the surface of PDA NPs by π-π interactions and the amino-quinone reaction, which can both increase mucus penetration and improve biocompatibility (Suk et al., 2016; Huckaby and Lai, 2018). LIP-1 was then added to the surface of PEG-modified PDA NPs through hydrogen bonding and π-π stacking interactions (Lu et al., 2019).

The size and morphology of PDA-PEG-LIP-1 NPs, PDA-PEG, and PDA were investigated using transmission electron microscopy (TEM). Well-dispersed spherical NPs were observed in all samples. The surface of PDA NPs was initially rough; however, after PEG modification and drug addition, the surface turned smooth (Figure 1B). The average diameters of PDA-PEG-LIP-1 NPs, PDA-PEG, and PDA were 180 nm, 205 nm, and 215 nm, respectively. The surface morphological features of the three types of NPs were analyzed via scanning electron microscopy (SEM). PDA-PEG NPs and PDA NPs were spherical with a smooth surface, whereas PDA-PEG-LIP-1 NPs exhibited additional white spots on their surface, which may be attributed to drug adsorption (Figure 1C).

The zeta potential of PDA-PEG-LIP-1 NPs, PDA-PEG, and PDA was −20.17 ± 0.94 mV, −24.48 ± 1.07 mV and −30.34 ± 1.98 mV, respectively, which validated the binding of PDA to the positively charged mNH_2_-PEG and/or LIP-1 (Figure 1D). The hydrodynamic sizes of the three types of NPs detected via dynamic light scattering (DLS) were 252.0 ± 5.0 nm, 260.2 ± 4.6 nm, and 267.9 ± 3.9 nm, respectively (Figure 1E). Numerous studies have demonstrated that nanoparticles with a 200–300 nm diameter can effectively reach the terminal airway without being cleared by macrophages (Guo et al., 2021; Deng et al., 2021). Consequently, the diameter of the PDA-PEG-LIP-1 NPs synthesized in this investigation was suitable. Figure 1F demonstrates that PDA-PEG-LIP-1 NPs and PDA-PEG were evenly distributed in ultrapure water. DLS measurements of particle size and PDI revealed no obvious changes in diameter and PDI within 7 days for PDA-PEG-LIP-1 NPs in simulated lung fluid at room temperature and 37 °C (Figures 1G–J), indicating excellent colloidal stability.

**FIGURE 2:**
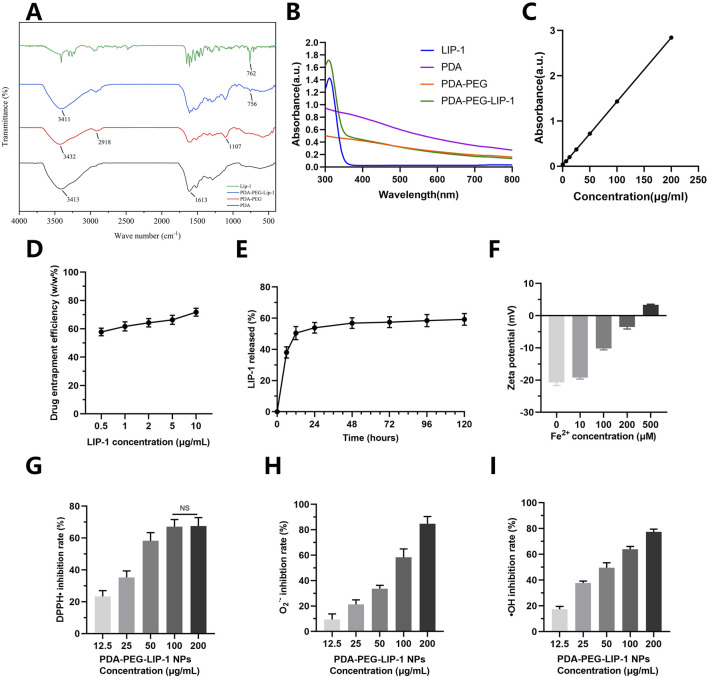
FT-IR spectra **(A)** and UV–vis spectra **(B)** of free LIP-1, PDA NPs, PDA-PEG NPs, and PDA-PEG-LIP-1 NPs (n = 5). **(C)** Calibration curve between the concentration of LIP-1 and its absorbance at 311 nm (n = 5). **(D)** The entrapment efficiency of PDA-PEG-LIP-1 NPs at various drug concentrations were measured via UV–vis spectroscopy (n = 5). **(E)** The releasing rate of LIP-1 from PDA-PEG-LIP-1 NPs at different time points was calculated via UV–vis spectroscopy (n = 5). **(F)** Zeta potential of PDA-PEG-LIP-1 NPs after 4 h of incubation with varied Fe^2+^ concentrations (n = 5). Scavenging activity of PDA-PEG-LIP-1 NPs for DPPH radical **(G)**, superoxide anion (O2·-) **(H)**, and hydroxyl radical (•OH) **(I)** (n = 4). Data are expressed as the mean ± SD (NS, no significant difference).

Using Fourier-transform infrared (FT-IR) spectroscopy, PEGylation and LIP-1 conjugation were further verified. As shown in Figure 2A, the O-H or N-H bond in PDA, the indole/benzene ring in PDA, the characteristic peaks at 2918 cm^-1^ and 1107 cm^-1^, the C-H bond and CH_2_CH_2_O in PEG, and the peak at 756 cm^-1^, the C-Cl bond in LIP-1 can all be related to the peak at 3413 cm^-1^. LIP-1 was effectively loaded on the surface of PDA-PEG NPs, as evidenced by the simultaneous appearance of the distinctive absorption peaks for PDA, PEG, and LIP-1.

The unique absorption peak LIP-1 at 311 nm was visible in the UV-vis absorption spectra of PDA-PEG-LIP-1 NPs (Figure 2B). PDA-PEG-LIP-1 NPs were quantified by measuring the intensity of this peak. In the concentration range of 0-200 μg/mL, a linear relationship between LIP-1 concentration and absorbance was detected. The regression equation was as follows: y = 0.0141x + 0.026 (R^2^ = 0.9999) (Figure 2C). As shown in Figure 2D, the entrapment efficiency of NPs could be altered by controlling the dosage of LIP-1. As the concentration of LIP-1 increased, the entrapment efficiency of NPs increased. The entrapment efficiencies at concentrations of 10, 5, 2, 1, and 0.5 μg/mL were approximately 57.8%, 61.8%, 64.3%, 66.4%, and 71.8%, respectively. According to these findings, the synthesized PDA-PEG NPs were effective drug carriers with a high drug entrapment efficiency and flexible control, which provided a good basis for subsequent investigations.

We used UV-vis spectroscopy to assess the release of LIP-1 from PDA-PEG-LIP-1 NPs in PBS *in vitro* at a pH value of 7.3, similar to the average airway pH (7.3) of asthma patients (Guo et al., 2021). The release rate of LIP-1 from PDA-PEG-LIP-1 NPs was approximately 53.8% after 24 h of incubation at 37 °C, whereas it was 59.2% at 120 h, indicating an increase of only 5.4% (Figure 2E). These results suggest that LIP-1 can be effectively released from PDA-PEG-LIP-1 NPs in the airway of asthma patients within 24 h of administration.

### 3.2 Iron-chelating and ROS-scavenging ability of PDA-PEG-LIP-1 NPs

Recent studies have reported that dysregulation of iron homeostasis and ferroptosis is associated with the development of asthma, suggesting that iron-chelating drugs may effectively treat neutrophilic asthma (Wientjens et al., 2025; Tang et al., 2026; Zhang et al., 2025). By evaluating their zeta potential, PDA-PEG-LIP-1 NPs were found to be effective at chelating Fe^2+^. Various quantities of Fe^2+^ were incubated with the PDA-PEG-LIP-1 solution for 4 h. The zeta potential of the solution increased from −20.72 to 3.35 mV as the Fe^2+^ concentration increased (Figure 2F), indicating that PDA-PEG-LIP-1 NPs chelated Fe^2+^ in a concentration-dependent manner.

We measured the clearance rates of hydroxyl radical (·OH), superoxide anion (O_2_^·-^), and DPPH radical to assess the ROS-scavenging capabilities of PDA-PEG-LIP-1 NPs. The efficacy of PDA-PEG-LIP-1 NPs in scavenging DPPH radicals improved as the concentration increased from 12.5 to 100 μg/mL, as demonstrated in Figure 2G. The DPPH radical-scavenging activity of 100 μg/mL PDA-PEG-LIP-1 NPs was 67.15% ± 3.88%, which was similar to that of 200 μg/mL PDA-PEG-LIP-1 NPs (67.55% ± 4.59%) (p > 0.05). As shown in Figure 2H, the O_2_^·-^-the scavenging ability of PDA-PEG-LIP-1 NPs increased with an increase in concentration. At concentrations of 100 and 200 μg/mL, the O_2_^·-^-scavenging activity of PDA-PEG-LIP-1 NPs was 58.33% ± 5.74% and 84.75% ± 4.92%, respectively. Figure 2I shows the ·OH-scavenging ability of PDA-PEG-LIP-1 NPs increased with their concentration. At concentrations of 100 and 200 μg/mL, the ·OH-scavenging ability of PDA-PEG-LIP-1 NPs was 63.98% ± 1.85% and 77.43% ± 1.81%, respectively. These findings indicate that the ROS-scavenging activity of PDA-PEG-LIP-1 NPs increased in a concentration-dependent manner and had a considerable scavenging effect on free radicals.

### 3.3 *In vitro* cytotoxicity and cellular uptake of PDA-PEG-LIP-1 NPs

**FIGURE 3:**
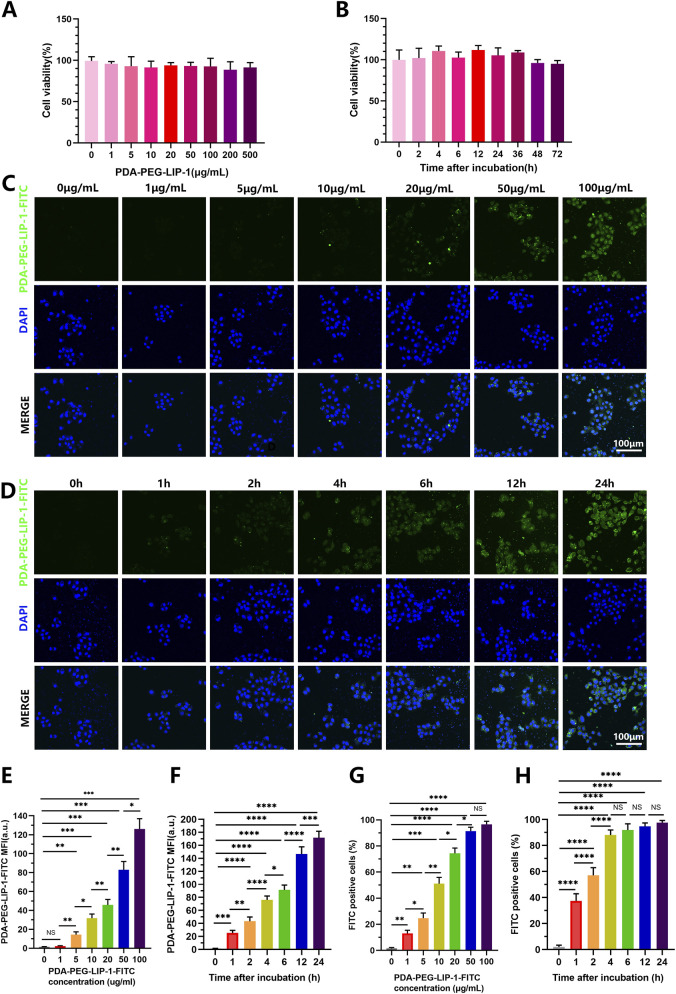
**(A)** PDA-PEG-LIP-1 NPs were used to treat HBE cells for 24 h at varied quantities. Cell viability was assessed using the CCK-8 test (n = 5). **(B)** HBE cells were treated with 100-μg/mL PDA-PEG-LIP-1 NPs at the indicated time points, and cell viability was detected via CCK-8 assay (n = 5). **(C)** Confocal laser scanning microscope (CLSM) images were taken of HBE cells incubated with different concentrations of PDA-PEG-LIP-1-FITC NPs for 4 h (magnification 400×, scale bar = 100 μm). **(D)** CLSM images of HBE cells incubated with PDA-PEG-LIP-1-FITC NPs (100 μg/mL) for different time points (magnification 400×, scale bar = 100 μm). **(E)** The Mean Fluorescence Intensity(MFI)of HBE cells treated with different concentrations of PDA-PEG-LIP-1-FITC nanoparticles for 4 h. **(F)** The MFI of HBE cells incubated with PDA-PEG-LIP-1-FITC NPs (100 μg/mL) for different time points. **(G,H)** Flow cytometry was performed to evaluate the number of FITC-positive HBE cells after treatment with PDA-PEG-LIP-1-FITC NPs. **(G)** PDA-PEG-LIP-1-FITC NPs were applied to cells at various doses for 4 h (n = 4). **(H)** Cells were treated with 100-μg/mL PDA-PEG-LIP-1-FITC NPs for different time points (n = 4). Data are expressed as the mean ± SD (*, P < 0.05; **, P < 0.01; ***, P < 0.001; ****, P < 0.0001; NS, no significant difference).

To evaluate the toxicity of PDA-PEG-LIP-1 NPs in HBE cells, the CCK-8 test was used. HBE cells showed no significant cytotoxicity when treated with PDA-PEG-LIP-1 NPs at concentrations between 0 and 500 μg/mL for 24 h (Figure 3A). Furthermore, treatment with 100 μg/mL PDA-PEG-LIP-1 NPs for 72 h had no discernible impact on the viability of HBE cells (Figure 3B).

To verify the abovementioned results, we assessed the uptake of FITC-labelled PDA-PEG-LIP-1 NPs in HBE cells. Cell nuclei were stained with DAPI. After 4 h of incubation, as illustrated in Figure 3C, the quantity of PDA-PEG-LIP-1-FITC NPs caused a steady rise in green fluorescence intensity. Green fluorescence was visible when PDA-PEG-LIP-1-FITC NP concentration reached 50 μg/mL. The results of mean fluorescence intensity were consistent with our observations (Figure 3E). Additionally, flow cytometry data revealed that the number of FITC-positive cells improved as the levels of PDA-PEG-LIP-1-FITC NPs increased (Figure 3G). Subsequently, HBE cells were incubated with 100 μg/mL PDA-PEG-LIP-1-FITC NPs at different time points (Figure 3D). The mean fluorescence intensity of PDA-PEG-LIP-1-FITC NPs increased with an increase in incubation time (Figure 3F). As the incubation time increases, the number of FITC-positive cells detected by flow cytometry also increases (Figure 3H). These observations indicate that PDA-PEG-LIP-1-FITC NPs were efficiently absorbed by HBE cells in a time- and concentration-dependent way.

### 3.4 PDA-PEG-LIP-1 NPs inhibited the production of ROS and lipid peroxides and exhibited iron-chelating ability and anti-ferroptosis effects in IL-13- and LPS-treated HBE cells

**FIGURE 4:**
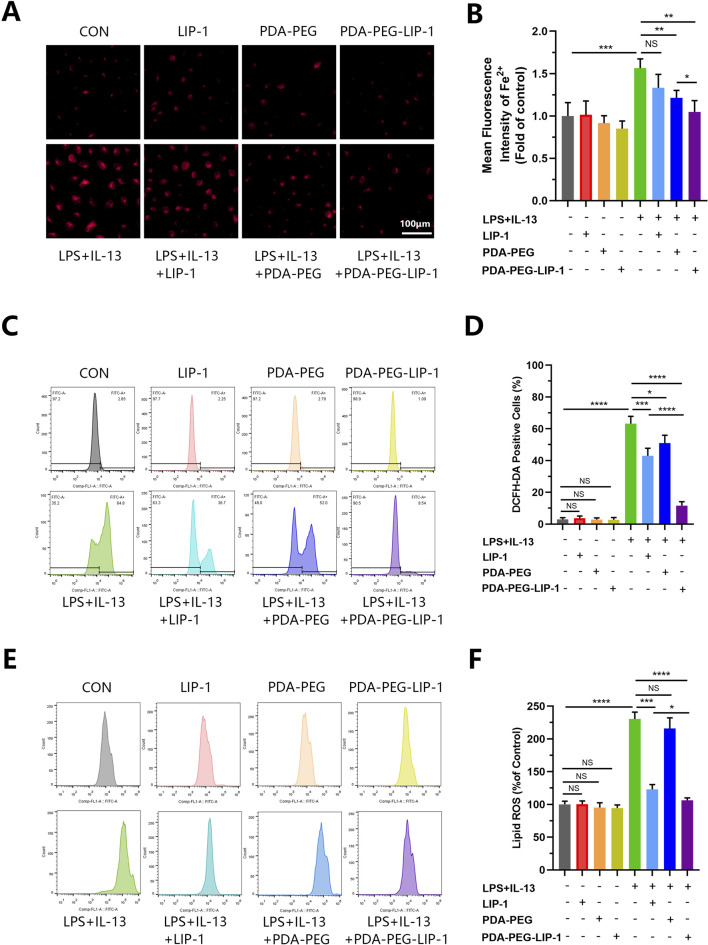
Illustrations images**(A)** for quantification **(B)** of free Fe^2+^ in HBE cells stained with FerroOrange (n = 4) (magnification 400×, scale bar = 100 μm). **(C,D)** Using DCFH-DA as the fluorescent probe, flow cytometry was used to identify ROS (n = 4). **(E,F)** Flow cytometry was applied to identify lipid ROS using the fluorescent probe C11-BODIPY (n = 4). Data are expressed as the mean ± SD (*, P < 0.05; **, P < 0.01; ***, P < 0.001; ****, P < 0.0001; NS, no significant difference).

HBE cells were treated with PBS, LIP-1, PDA-PEG NPs, or PDA-PEG-LIP-1 NPs and incubated with or without LPS and IL-13 for 24 h to test the anti-ferroptosis effects of PDA-PEG-LIP-1 NPs. The iron-chelating capacity of PDA-PEG-LIP-1 NPs was first evaluated. FerroOrange was used to detect intracellular free Fe^2+^ in HBE cells. Specifically, FerroOrange reacts with Fe^2+^ to form a substance that produces vibrant orange fluorescence at 590 nm (Tian et al., 2021). Figure 4A shows that LPS and IL-13 treatment significantly increased the fluorescence signals in HBE cells. The lighter fluorescence signals displayed by the cells in the LPS + IL-13 + PDA-PEG NP and LPS + IL-13 + PDA-PEG-LIP-1 NP groups suggested lower amounts of free Fe^2+^. These findings disclose that PDA-PEG-LIP-1 NPs efficiently chelated free Fe^2+^ in LPS- and IL-13-treated HBE cells (Figure 4B).

Excessive deposition of intracellular free Fe^2+^ triggers the Fenton reaction to produce ROS, which induces cellular lipid peroxidation. ROS are significant mediators in the development of asthma and are responsible for airway remodeling, hyperresponsiveness, and inflammation (Michaeloudes et al., 2022). LPS and IL-13 can stimulate ROS secretion from HBE cells to mimic the oxidative stress and inflammatory microenvironment of neutrophilic asthma *in vitro* (Bao et al., 2022). In this study, ROS released from LPS- and IL-13-treated HBE cells were detected via flow cytometry using DCFH-DA as the fluorescent probe. PDA-PEG-LIP-1 NPs at a concentration of 100 μg/mL reduced ROS formation in HBE cells treated with IL-13 and LPS compared with LPS and PDA-PEG NPs (Figures 4C,D).

Furthermore, the fluorescent probe C11-BODIPY was used to determine the levels of lipid peroxides in IL-13- and LPS-treated HBE cells. The results revealed that both LIP-1 and PDA-PEG-LIP-1 NPs inhibited the production of lipid peroxides, with PDA-PEG-LIP-1 NPs showing stronger effects than LIP-1 (Figures 4E,F). Lipid peroxidation is the key downstream factor of ferroptosis. Elevated levels of lipid peroxides have been reported in patients with asthma (Alzoghaibi and Bahammam, 2007). Therefore, we speculate that PDA-PEG-LIP-1 NPs can inhibit ferroptosis by suppressing cellular lipid peroxidation.

**FIGURE 5:**
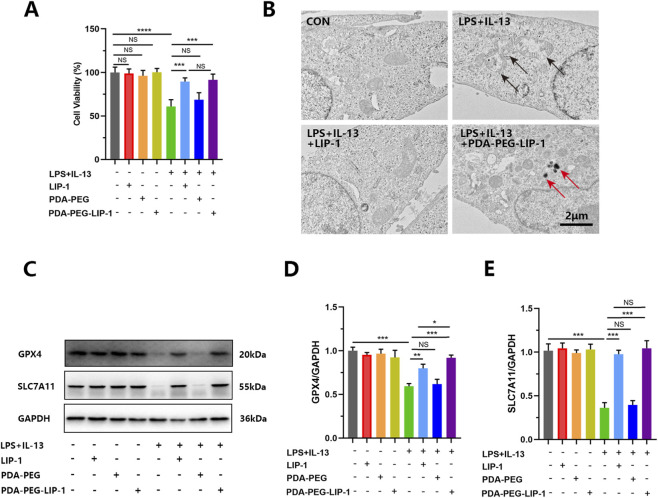
**(A)** CCK-8 assay was performed to assess the viability of HBE cells in different groups (n = 5). **(B)** Illustration of TEM images of HBE cells following various treatments. Black arrows indicate the damaged mitochondria, whereas red arrows denote PDA-PEG-LIP-1 NPs internalized by the cells (magnification 5000×, scale bar = 2 μm). **(C)** Protein expression of GPX4 and SLC7A11 in HBE cells after different treatments. Expression of GPX4 **(D)** and SLC7A11 **(E)** in the specified groups (n = 3). Data are expressed as the mean ± SD (*, P < 0.05; **, P < 0.01; ***, P < 0.001; ****, P < 0.0001; NS, no significant difference).

According to the results of the CCK-8 assay, LPS and IL-13 significantly decreased the viability of HBE cells, whereas both LIP-1 and PDA-PEG-LIP-1 effectively restored cell viability (Figure 5A). TEM demonstrated aberrant mitochondrial morphology in the IL-13 + LPS group, characterized by fragmented mitochondrial cristae, swelling, and disappearance (black arrows). Fewer morphological changes were observed in the LIP-1 + IL-13 + LPS group than in the IL-13 + LPS group. In the PDA-PEG-LIP-1 NP + IL-13 + LPS group, nanoparticles (red arrows) were visible in the cytoplasm of cells, and the morphology of mitochondria was almost normal (Figure 5B).

In addition, the expression levels of the ferroptosis-related proteins GPX4 and SLC7A11 were assessed. GPX4 and SLC7A11 expression was lower in the LPS + IL-13 group compared with the control (CON) group. Both LIP-1 and PDA-PEG-LIP-1 NPs effectively restored SLC7A11 expression, with PDA-PEG-LIP-1 NPs being more effective in restoring GPX4 expression in LPS- and IL-13-treated cells (Figures 5C–E).

These results indicate that PDA-PEG-LIP-1 NPs protected HBE cells from ferroptosis by reducing cellular lipid peroxidation and ROS production, chelating intracellular free Fe^2+^, and restoring the expression of ferroptosis-related proteins.

### 3.5 *In vivo* uptake and biocompatibility of PDA-PEG-LIP-1 NPs

**FIGURE 6:**
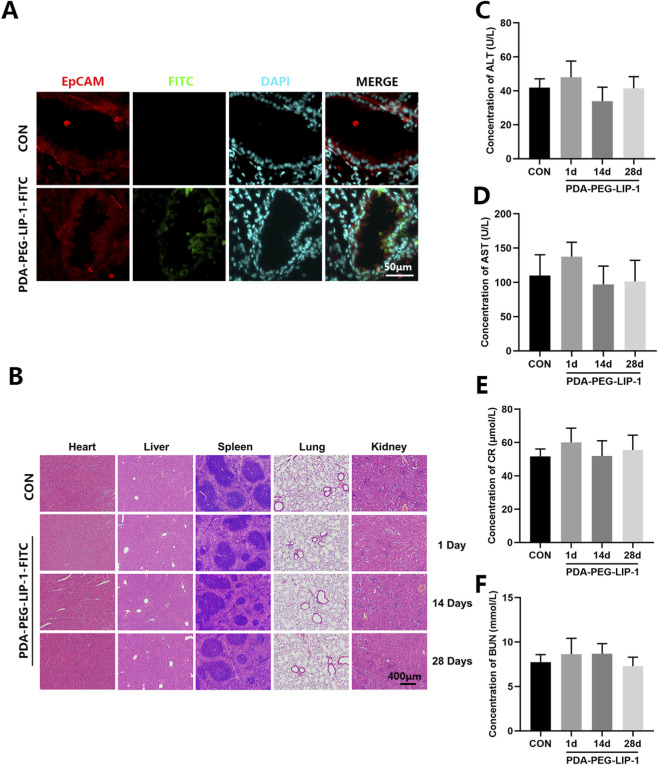
**(A)** CLSM images of PDA-PEG-LIP-1-FITC NPs in the lung sections of mice. Cy3-labeled anti-EpCAM antibodies (red), FITC-labelled PDA-PEG-LIP-1 NPs (green), and DAPI-labelled nuclei (blue) (magnification 400×, scale bar = 50 μm). **(B)** H&E staining of the main organs of mice treated with 100-μg/mL PDA-PEG-LIP-1 NPs after 1 day, 14 days, and 28 days of inhalation (magnification 100×, scale bar = 400 μm). Levels of alanine transaminase (ALT) **(C)**, aspartate transaminase (AST) **(D)**, creatinine (CR) **(E)**, and blood urea nitrogen (BUN) **(F)** in mouse serum (n = 5).

FITC-labeled PDA-PEG-LIP-1 NPs were administered via intranasal (i.n.) instillation to the airways of mice to evaluate NP uptake by mouse airway epithelial cells. Confocal laser scanning microscopy (CLSM) revealed that the NPs were effectively internalized by epithelial cells within 24 h of i. n. Instillation (Figure 6A).

After 7, 14, and 28 days of i. n, instillation of 10-mg/kg PDA-PEG-LIP-1 NPs, hematoxylin and eosin (H&E) staining of the kidneys, lungs, hearts, spleens, and livers of mice showed no significant necrosis, inflammation, hemorrhage or other injuries (Figure 6B). Additionally, there were no changes in the mice’s serum levels of the liver and kidney function markers aspartate transaminase (AST), alanine transaminase (ALT), creatinine (CR), and blood urea nitrogen (BUN) (Figures 6C–F). According to these findings, PDA-PEG-LIP-1 NPs displayed excellent safety *in vivo* with only mild toxicity.

### 3.6 By preventing ferroptosis, PDA-PEG-LIP-1 NPs reduced neutrophilic asthma in mice

**FIGURE 7:**
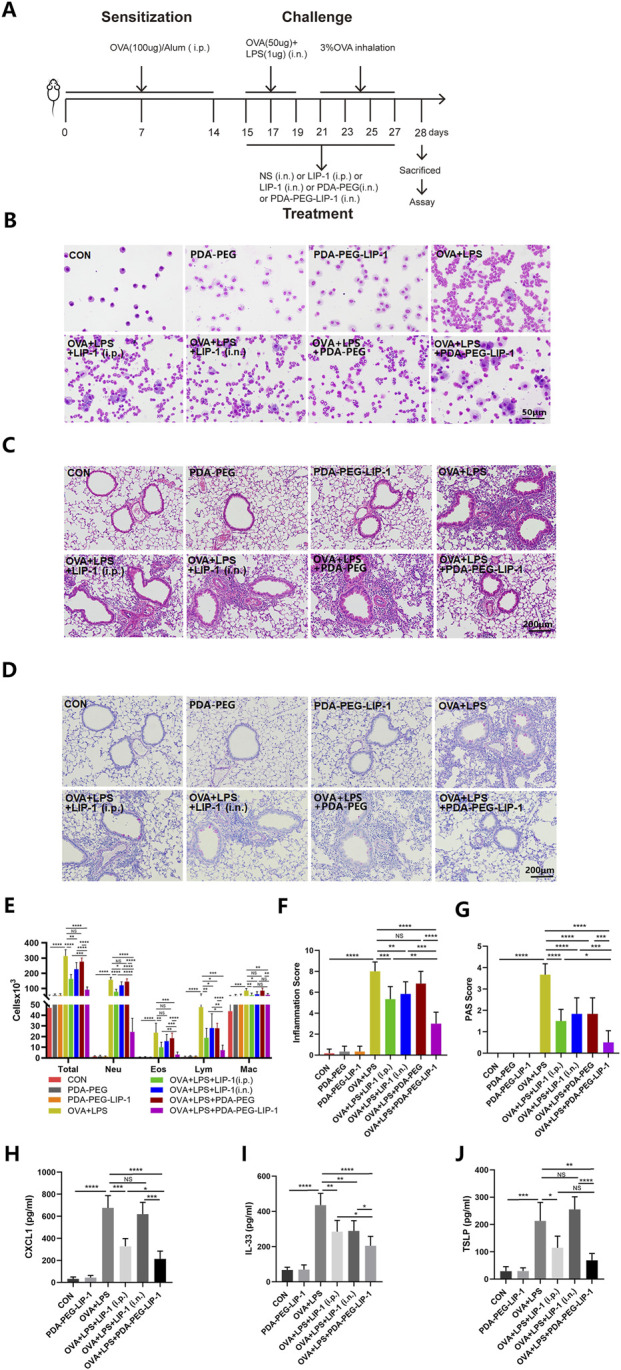
**(A)** Flow chart of experiments on mouse models. **(B)** Liu’s staining of mouse bronchoalveolar lavage fluid (BALF) in the indicated groups (magnification 400×, scale bar = 50 μm). Lung sections stained with H&E **(C)** and PAS **(D)** in the specified groups (×200 magnification, 200 μm scale bar). **(E)** Statistical analysis of the number of macrophages, neutrophils, lymphocytes, and eosinophils in BALF (n = 6). Using H&E scores (n = 6), inflammatory cell infiltration was quantified **(F)**. **(G)** Quantification of goblet cell hyperplasia was based on PAS scores (n = 6). ELISA was performed to detect and evaluate the concentration of CXCL1 **(H)**, IL-33 **(I)**, and TSLP **(J)** in BALF in the indicated groups of mice. Data are expressed as the mean ± SD (*, P < 0.05; **, P < 0.01; ***, P < 0.001; ****, P < 0.0001; NS, no significant difference).

Due to the effects of anti-ferroptosis activity of PDA-PEG-LIP-1 NPs observed *in vitro*, we hypothesized whether PDA-PEG-LIP-1 NPs could alleviate OVA- and LPS-induced neutrophilic asthma by inhibiting ferroptosis *in vivo*. Mouse models of asthma were established, as shown in Figure 7A. According to Liu’s staining of mouse bronchoalveolar lavage fluid (BALF), the OVA + LPS group had significantly higher numbers of neutrophils and eosinophils, which is consistent with the phenotype of neutrophilic asthma (Figures 7B,E). The OVA + LPS + LIP-1 (intraperitoneal [i.p.] injection or i. n. instillation) group and OVA + LPS + PDA-PEG group considerably exceeded the OVA + LPS + PDA-PEG-LIP-1 NPs group in terms of both the total inflammatory cell count and numbers of neutrophils, eosinophils, macrophages, and lymphocytes in BALF (Figures 7B,E).

Subsequently, we compared the results of H&E (Figure 7C) and PAS (Figure 7D) staining of lung tissue sections among different groups and evaluated inflammation scores. Conventionally, compared with CON mice sensitized to saline, OVA- and LPS-treated mice exhibited high inflammatory cell infiltration in bronchioles, thickened airway epithelium, and increased numbers of goblet cells and airway mucus. Three treatments (LIP-1 via i. p. Injection, LIP-1 via i. n. Instillation, and PDA-PEG-LIP-1 NPs via i. n. instillation) alleviated OVA- and LPS-induced peritracheal inflammation and goblet cell hyperplasia and reduced mucus secretion to some extent. The PDA-PEG NPs treatment ameliorated goblet cell hyperplasia and airway mucus hypersecretion alone, with no obvious improvement in peritracheal inflammation. However, PDA-PEG-LIP-1 NPs administered via i. n. Instillation were the most effective (Figures 7F,G). TSLP, CXCL1, and IL-33 are inflammatory factors released by airway epithelial cells during the development of asthma (Hammad and Lambrecht, 2021). ELISA showed that the concentrations of TSLP, CXCL1, and IL-33 in BALF were lower in the OVA + LPS + PDA-PEG-LIP-1 NP group than in the OVA + LPS + LIP-1 (i.p. Injection or i. n. instillation) group (Figures 7H–J). Overall, our findings revealed that in asthmatic mice, PDA-PEG-LIP-1 NPs administered via intranasal instillation were superior to LIP-1 administered via intranasal or intraperitoneal injection in reducing mucus secretion, airway inflammation, and proinflammatory cytokine release.

**FIGURE 8:**
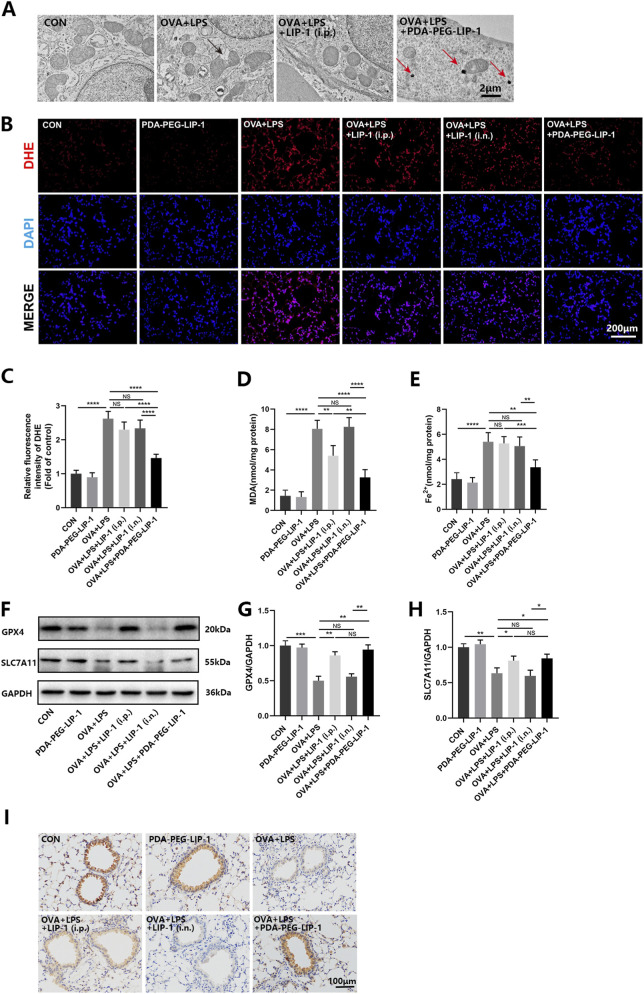
**(A)** After various procedures, representative TEM photographs of mouse lung epithelial cells were captured. Black arrows indicate damaged mitochondria, whereas red arrows indicate PDA-PEG-LIP-1 NPs internalized by the cells (magnification 5000×, scale bar = 2 μm). **(B)** Fluorescent images of dihydroethidium (DHE)-stained mouse lung sections (magnification 200×, scale bar = 200 μm). **(C)** Comparative analysis for DHE staining. The relative concentration of MDA **(D)** and Fe^2+^ **(E)** in mouse lung tissues was measured (n = 5). **(F)** Protein expression of SLC7A11 and GPX4 in mouse lung tissues after different treatments. The protein expression of GPX4 **(G)** and SLC7A11 **(H)** was quantified in the indicated groups (n = 3). **(I)** Images of representative immunohistochemical GPX4 staining in mice lung sections (magnification 400×, scale bar = 100 μm). Data are expressed as the mean ± SD (*, P < 0.05; **, P < 0.01; ***, P < 0.001; ****, P < 0.0001; NS, no significant difference).

In addition, we explored the mechanism underlying the therapeutic effects of PDA-PEG-LIP-1 NPs against neutrophilic asthma. TEM revealed the presence of ruptured mitochondria (black arrow) in airway epithelial cells in the OVA + LPS group. However, treatment with PDA-PEG-LIP-1 NPs (red arrows) rescued mitochondrial damage (Figure 8A). ROS generation in lung tissues was more substantial in the OVA + LPS group than in the CON group, according to dihydroethidium (DHE) staining. In addition, administration of PDA-PEG-LIP-1 NPs via i. n. Instillation significantly inhibited ROS production (Figures 8B,C). MDA or Fe^2+^ levels in lung tissues were higher in the OVA + LPS group than in the CON group. Both PDA-PEG-LIP-1 NPs administered via i. n. Instillation and LIP-1 administered via i. p. Injection reduced MDA levels. However, PDA-PEG-LIP-1 NPs were more effective in reducing MDA and Fe^2+^ levels (Figures 8D,E). Western blotting revealed that treatment with OVA and LPS reduced the protein expression of SLC7A11 and GPX4 in lung tissues, whereas administration of PDA-PEG-LIP-1 NPs via i. n. Instillation effectively restored the protein expression of both factors (Figures 8F–H). The immunohistochemical staining results were similar to western blotting for GPX4 (Figure 8I). Altogether, these results indicated that inhalation of PDA-PEG-LIP-1 NPs relieved neutrophilic asthma by inhibiting ferroptosis in airway epithelial cells in mice.

**FIGURE 9:**
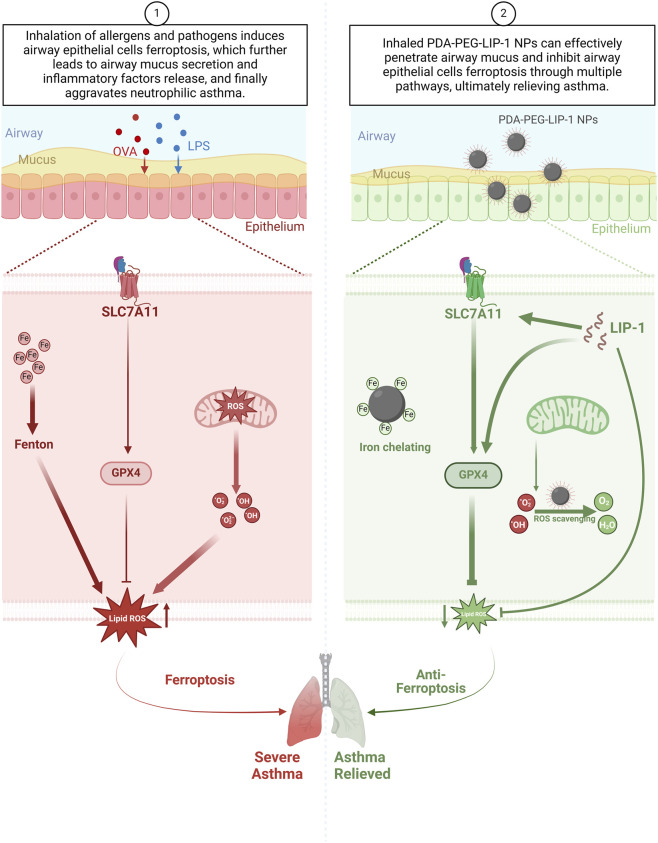
Representation of the PDA-PEG-LIP-1 NPs’ therapeutic mode of action in mice with LPS- and OVA-induced asthma.

The potential mechanism of action of PDA-PEG-LIP-1 NPs in the treatment of neutrophilic asthma is summarised in Figure 9. Inhalation of allergens and pathogens induces ferroptosis in airway epithelial cells, leading to airway mucus secretion and inflammatory factor release and eventually aggravating neutrophilic asthma. Inhaled PDA-PEG-LIP-1 NPs can effectively penetrate airway mucus and inhibit the ferroptosis of airway epithelial cells through multiple pathways, ultimately alleviating neutrophilic asthma.

## 4 Discussion

Neutrophilic asthma is a chronic airway inflammatory disease involving multiple cells, cytokines, and inflammatory mediators. This type of asthma responds poorly to conventional glucocorticoid therapy, and its pathogenesis has not been fully elucidated yet, which is a major obstacle to its clinical treatment (Hammad et al., 2025). Conventional pharmacological treatments can only control asthmatic symptoms and cannot reverse the natural disease progression. Therefore, further exploration of new anti-inflammatory drugs and delivery routes has always been a research focus in the field of asthma treatment.

Due to the thin alveolar-capillary barrier and large surface area of the lung, administration of medication via inhalation offers numerous advantages over conventional delivery routes. It offers low enzymatic exposure, fewer systemic side effects, avoidance of first-pass metabolism, and high local drug concentrations at the disease site, making it an ideal route for the treatment of pulmonary diseases (Banat et al., 2023).

Airway epithelial cells play a vital role in the occurrence and development of asthma, which is why they are one of the target cells for asthma treatment (Hellings and Steelant, 2020). Ferroptosis is an iron-dependent form of programmed cell death triggered by lipid peroxidation. Excessive accumulation of iron ions and ROS, as well as decreased activities of GPX4 and SLC7A11, have been confirmed to induce cellular ferroptosis (Dixon and Olzmann, 2024). Recent studies have suggested that ferroptosis in airway epithelial cells is closely associated with the occurrence of asthma (Ni et al., 2025; Yin et al., 2025). Our previous studies verified the potential of LIP-1 in treating neutrophilic asthma by inhibiting ferroptosis (Bao et al., 2022).

In this study, the human bronchial epithelial cell line (HBE cells) was treated with LPS combined with IL-13 to simulate the microenvironment of neutrophilic asthma (Bao et al., 2022). The synthesized PDA-PEG-LIP-1 nanoparticles were confirmed to improve cell viability. The PEG moiety of nanoparticles facilitate penetration through the mucus layer in the asthmatic airway. After internalization into the airway epithelial cells, the nanoparticles release LIP-1 to regulate the SLC7A11 and GPX4 pathways. In addition, the PDA component can chelate excessive Fe^2+^ in cells, reduce the production of reactive oxygen species (ROS), protect mitochondrial function, and jointly reduce cell ferroptosis and the secretion of pro-inflammatory cytokines. This nanoparticle drug delivery system can enhance the water solubility of LIP-1, while increasing the release and retention of the drug in the respiratory tract, thereby guaranteeing therapeutic efficacy and improving its biocompatibility.

More importantly, CXCL1, IL-33, and thymic stromal lymphopoietin (TSLP) are predominantly secreted by airway epithelial cells. These cytokines drive the production of downstream Th2-type cytokines, and are thus closely associated with the severity of asthma (Lambrecht et al., 2019). Intratracheal inhalation of PDA-PEG-LIP-1 nanoparticles markedly inhibits ferroptosis in airway epithelial cells, reduces the secretion of pro-inflammatory cytokines including CXCL1, IL-33, and TSLP, and attenuates airway inflammation and mucus hypersecretion in asthmatic mice, ultimately alleviating asthmatic symptoms.

Previous studies have loaded small-molecule drugs or nucleic acids into nanoparticles composed of various materials such as lipids and polymers for targeted therapy of asthma, confirming the effectiveness and advantages of nanoparticle-mediated targeting strategies (Zhang et al., 2022; Roy et al., 2020). However, existing nanoparticles have not been investigated or applied in the regulation of ferroptosis and mitochondrial protection in airway epithelial cells in neutrophilic asthma, and their regulatory effects on asthmatic airway epithelial cells are limited. In addition, the anti-asthmatic activity of such nanoparticles mainly relies on the loaded therapeutic drugs, and the nanocarriers and small-molecule drugs do not exert synergistic effects in inhibiting cell death. Meanwhile, the preparation procedures of drug-loaded nanoparticles are usually complicated. Importantly, the therapeutic efficacy of such nanoparticles against neutrophilic asthma still needs to be verified. In contrast, the PDA-PEG-LIP-1 nanoparticles prepared in this study are constructed based on safe and reliable biomaterials. The nanocarrier PDA and small-molecule drug LIP-1 can synergistically inhibit cellular ferroptosis and protect mitochondria through multiple molecular mechanisms. Furthermore, they feature simple preparation, controllable quality, and low cost, thus exhibiting favorable translational potential and enabling precision therapy for neutrophilic asthma.

This research has several limitations. Methodologically, only HBE cells were used, and primary cells were not employed to simulate the asthmatic airway microenvironment. Regarding the methods of investigation, the conclusions on ferroptosis inhibition were mostly based on correlation analysis, and functional experiments such as gene knockout were lacking to verify the specific roles of the relevant targets. In terms of experimental detection, the systemic biodistribution and clearance of inhaled nanoparticles were not investigated, leaving potential off-target accumulation unaddressed. Future studies will focus on further validating ferroptosis-related pathways and integrating multi-omics technologies to comprehensively elucidate the systematic action mechanism of PDA-PEG-LIP1 NPs.

## 5 Conclusion

PDA-PEG-LIP-1 NPs developed in this study effectively alleviate neutrophilic asthma in a female mouse model due to their superior antioxidant, anti-ferroptosis, and anti-inflammatory properties. These NPs can effectively deliver LIP-1 to airway epithelial target cells, which improves drug delivery efficiency and practicability. Meanwhile, they enhance the safety of LIP-1 by eliminating adverse reactions caused by systemic administration. PDA-PEG-LIP-1 NPs are capable of chelating Fe^2+^, upregulating the protein expression of GPX4 and SLC7A11, and reducing ROS production as well as lipid peroxidation, thereby suppressing ferroptosis and the secretion of inflammatory cytokines. In the future, further validations are needed in various cell lines and animal models, and the experimental detection should be better optimized and potential sex differences in the effects of NPs should be addressed. Collectively, the present research reveals the promising potential of nanomedicine for the treatment of neutrophilic asthma.

## Data Availability

The original contributions presented in the study are publicly available. This data can be found here: https://doi.org/10.57760/sciencedb.37221.
